# Estrogen therapy after breast cancer diagnosis and breast cancer mortality risk

**DOI:** 10.1007/s10549-023-06871-w

**Published:** 2023-02-11

**Authors:** Maria Sund, Hans Garmo, Anne Andersson, Sara Margolin, Johan Ahlgren, Antonis Valachis

**Affiliations:** 1 Department of Oncology, Faculty of Medicine and Health, Örebro University Hospital, Örebro University, 70182 Örebro, Sweden; 2 Sweden Regional Cancer Center, Uppsala University Hospital, Uppsala University, Uppsala, Sweden; 3 Department of Radiation Sciences, Oncology, Umeå University, Umeå, Sweden; 4 Department of Oncology, Södersjukhuset, Stockholm, Sweden; 5 Department of Clinical Science and Education, Södersjukhuset, Karolinska Institutet, Stockholm, Sweden

**Keywords:** Breast cancer, Estrogen therapy, Survivors, Endocrine therapy

## Abstract

**Purpose:**

The safety of local estrogen therapy in patients on adjuvant endocrine treatment is questioned, but evidence on the issue is scarce. This nested case–control registry-based study aimed to investigate whether estrogen therapy affects breast cancer mortality risk in women on adjuvant endocrine treatment.

**Methods:**

In a cohort of 15,198 women diagnosed with early hormone receptor (HR)-positive breast cancer and adjuvant endocrine treatment, 1262 women died due to breast cancer and were identified as cases. Each case was matched with 10 controls. Exposure to estrogen therapy with concurrent use of aromatase inhibitors (AIs), tamoxifen, or both sequentially, was compared between cases and controls.

**Results:**

No statistically significant difference in breast cancer mortality risk was seen in patients with exposure to estrogen therapy concurrent to endocrine treatment, neither in short-term or in long-term estrogen therapy use.

**Conclusions:**

The study strengthens current evidence on local estrogen therapy use in breast cancer survivors, showing no increased risk for breast cancer mortality in patients on adjuvant AIs or tamoxifen.

## Introduction

Adjuvant endocrine treatment has shown to improve survival in breast cancer patients with hormone-receptor (HR)-positive disease, with tamoxifen being the treatment of choice for premenopausal women whereas AIs are preferred options in postmenopausal women [1, 2]. However, the adherence to endocrine treatment is considerably low, mainly due to the presence of side effects influencing patients’ quality of life [3, 4]. Poor adherence to endocrine treatment impacts the prognosis [5].

A common side effect of endocrine treatment, with possible effects on adherence, is genitourinary symptoms due to vaginal atrophy [6–8]. The treatment strategy for women without prior breast cancer suffering from vaginal atrophy is based on the use of systemic and local estrogen therapy. In patients with prior breast cancer, use of systemic estrogen therapy is not recommended, based on three randomized controlled trials investigating estrogen replacement therapy in breast cancer survivors that stopped early due to increased risk for development of a new breast cancer or recurrence in two of them [9–11].

Local estrogen therapy can improve genitourinary symptoms [12]. However, the safety of local estrogen therapy in breast cancer patients has been questioned based on some small prospective studies which found increased blood hormone levels when local estrogen therapy was used in patients with adjuvant endocrine treatment [13–15]. Whether the increased blood hormone levels could be translated to a clinically relevant increase in risk for recurrence or mortality due to breast cancer is largely unknown. Two observational studies investigating the potential impact of local estrogen therapy on breast cancer recurrence have been published so far without indications on increased risk for patients using local therapy [16, 17]. However, these studies are prone to bias due to small sample size [16] as well as lacking power to study AI users separately [17], thus, making the interpretation of results questionable.

The purpose of the present study was to investigate the impact of local estrogen therapy in HR-positive breast cancer patients with adjuvant endocrine treatment on breast cancer mortality and whether the duration of exposure to estrogen therapy or the type of adjuvant endocrine treatment could influence the potential association.

## Methods

### Data source

For this study, the research database Breast Cancer Database Sweden (BCBaSe) was used. BCBaSe is a linkage between breast cancer quality registers and several other population-based registries, with information on individuals diagnosed with breast cancer between 1992 and 2012 in three Swedish health care regions; Stockholm-Gotland, Uppsala-Orebro and the Northern health care regions, comprising approximately 50% of the Swedish population.

The breast cancer quality registers hold information on tumor characteristics, treatment, and follow-up [18, 19]. Population-based registers with linkage in BCBaSe include the Cause of Death Register, with information on date of death and underlying cause of death [20], the Prescribed Drug Register, with information on all prescribed medications filled in Swedish pharmacies with information regarding defined daily doses (DDD) [21, 22], Longitudinal Integration Database for Health Insurance and Labour Market Studies (LISA), with data on socioeconomic variables such as marital status, education level and income [23], as well as the National Patient Register with information on diagnoses in hospital care [24, 25]. By combining relevant data from BCBaSe, the Charlson Comorbidity Index (CCI) as well as a Drug Comorbidity Index (DCI) for each patient was calculated. Both CCI and DCI have prognostic value in terms of overall survival in cancer patients [26, 27]. BCBaSe is previously described in detail [28].

### Study cohort

**Fig. 1:**
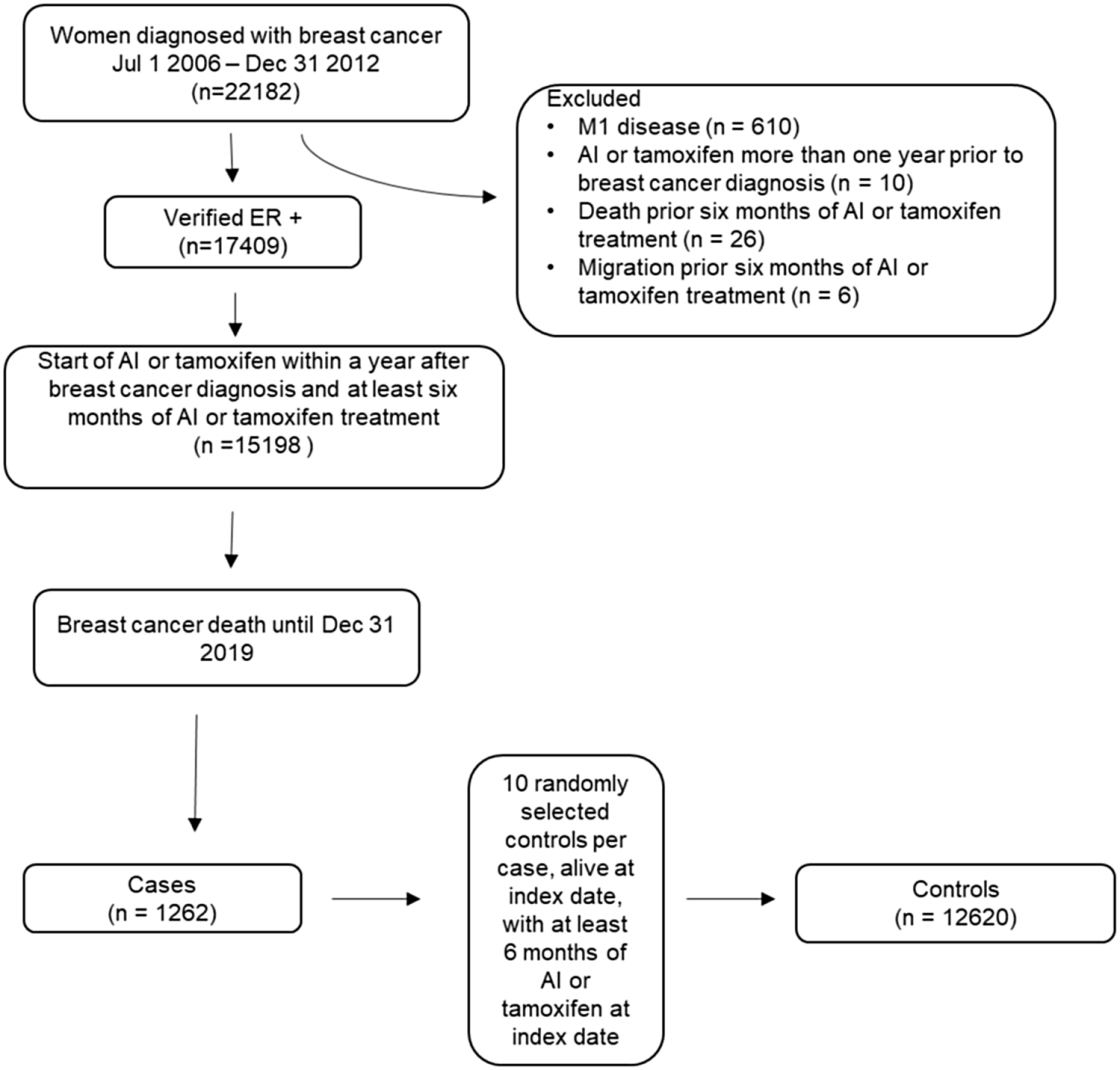
Flowchart diagram of study cohort

The study cohort included women from BCBaSe diagnosed with HR-positive breast cancer between July 1, 2006 and December 31, 2012, with at least six months of treatment with AIs or tamoxifen in total and with a filled prescription of AIs or tamoxifen within 1 year after breast cancer diagnosis. We excluded patients with distant metastases at diagnosis, patients who emigrated or died before six months of endocrine treatment, as well as patients who had filled prescription of AIs or tamoxifen more than 1 year prior to breast cancer diagnosis. Last day of follow-up was December 31, 2019. See flowchart in Fig. 1.

### Study design

The study was conducted with nested case–control design. Patients in the study cohort who died due to breast cancer before end of follow-up were selected as cases, with date of breast cancer-related death serving as index date. By incidence density sampling, each case was matched with 10 randomly selected controls from the study cohort, alive, and at risk at index date.

### Exposure to estrogen therapy

Exposure to estrogen therapy was defined as at least one filled prescription of either estriol (Anatomical Therapeutic Chemical (ATC) code G03CA04) or estradiol (ATC code G03CA03), at least 1 year before index date. Exposure to estrogen therapy was compared regarding concurrent use of AI, tamoxifen, or both, as well as no concurrent use of endocrine treatment. Concurrent use was defined as a filled prescription of AI or tamoxifen within 90 days before or after a filled prescription of estrogen therapy. No exposure to estrogen therapy served as reference.

### Short- and long-term estrogen exposure

To investigate the potential impact of estrogen therapy exposure on breast cancer mortality, the study cohort exposed to estrogen therapy was divided into two groups, based on DDD of estrogen therapy. Short -term exposure was defined as a DDD of estrogen therapy less than the 66th percentile of the total estrogen therapy exposed patient cohort, whereas long-term exposure was defined as a DDD of more than the 66th percentile. The data-driven cut-off value of DDD for defining short- or long-term exposure was 90 days.

### Statistical analysis

Conditional logistic regression analyses were used for estimation of Odds Ratio (OR) and 95% Confidence Interval (CI) concerning exposure in uni- and multivariate analyses. The multivariate analysis was performed in two steps: first including potential confounders related to tumor characteristics and patients’ age; age at breast cancer diagnosis, time from breast cancer diagnosis to index date, tumor stage, nodal stage, pre- or postoperative chemotherapy. The second step also included covariates related to patient characteristics; family income, marital status, educational level, previous estradiol or estriol treatment more than 1 year before breast cancer diagnosis, DCI, CCI, and health care region.

All analyses were performed on R version R version 4.1.2 ("Bird Hippie" Copyright (C) 2021 The R Foundation for Statistical Computing). Statistical significance was considered when 95% CI did not include the value of 1.

## Results

### Study cohort

During follow-up, 1262 women in the cohort died due to breast cancer and were identified as cases. All cases were matched with 10 controls each, resulting in 12,620 controls. Median time from breast cancer diagnosis to index date was 5.1 years (range: 0.4–13.3 years). The characteristics of cases and controls are presented in Table 1. Cases comprised older patients, with higher T- and N-stage, more frequently treated with chemotherapy and AIs, higher DCI and CCI scores, as well as a lower socioeconomic status.

**Table 1:** Characteristics of cases and controls

	Cases (*n* = 1262)	Controls (*n* = 12,620)	OR	(95% CI)
Age, *n* (%)				
≤ 50	234 (18.5)	2891 (22.9)	1.00	1
51–60	256 (20.3)	2919 (23.1)	1.08	(0.90–1.30)
61–70	310 (24.6)	4155 (32.9)	0.92	(0.77–1.10)
71–80	256 (20.3)	1874 (14.8)	1.70	(1.41–2.04)
> 80	206 (16.3)	781 (6.2)	3.32	(2.71–4.08)
Health care region				
Stockholm/Gotland	531 (42.1)	5908 (46.8)	1.00	1
Uppsala/Örebro	529 (41.9)	5005 (39.7)	1.18	(1.04–1.33)
Northern	202 (16.0)	1707 (13.5)	1.32	(1.11–1.56)
T stage				
T1	220 (17.4)	6821 (54.0)	1.00	1
T2	772 (61.2)	4961 (39.3)	4.90	(4.20–5.72)
T3	190 (15.1)	648 (5.1)	9.07	(7.35–11.2)
T4	62 (4.9)	123 (1.0)	15.92	(11.4–22.3)
TX	18 (1.4)	67 (0.5)	8.37	(4.88–14.34)
N stage				
N0	414 (32.8)	8297 (65.7)	1.00	1
N +	823 (65.2)	4280 (33.9)	3.86	(3.41–4.37)
Missing	25 (2.0)	43 (0.3)	12.39	(7.39–20.77)
Chemotherapy				
No chemotherapy	584 (46.3)	7855 (62.2)	1.00	1
Chemotherapy	678 (53.7)	4765 (37.8)	1.93	(1.72–2.17)
Tamoxifen use				
No tamoxifen	363 (28.8)	3316 (26.3)	1.00	1
0–2 years	503 (39.9)	3354 (26.6)	1.41	(1.22–1.63)
2–5 years	320 (25.4)	3879 (30.7)	0.74	(0.63–0.86)
5 + years	76 (6.0)	2071 (16.4)	0.30	(0.23–0.39)
AI use				
No AI	137 (10.9)	5549 (44.0)	1.00	1
0–2 years	506 (40.1)	2777 (22.0)	7.51	(6.17–9.14)
2–5 years	463 (36.7)	3122 (24.7)	6.18	(5.06–7.53)
5 + years	156 (12.4)	1172 (9.3)	5.56	(4.34–7.11)
Marital status				
Married	557 (44.1)	6485 (51.4)	1.00	1
Not married	705 (55.9)	6135 (48.6)	1.34	(1.19–1.50)
Family income				
Low	571 (45.2)	4017 (31.8)	1.00	1
Intermediate	397 (31.5)	4189 (33.2)	0.66	(0.58–0.76)
High	278 (22.0)	4304 (34.1)	0.45	(0.39–0.52)
Missing	16 (1.3)	110 (0.9)	1.01	(0.60–1.72)
Educational level				
Low	403 (31.9)	2897 (23.0)	1.00	1
Intermediate	496 (39.3)	5178 (41.0)	0.69	(0.60–0.79)
High	363 (28.8)	4545 (36.0)	0.57	(0.49–0.67)
Drug Comorbidity Index (quartile)				
1 (lowest)	173 (13.7)	3299 (26.1)	1.00	1
2	244 (19.3)	3225 (25.6)	1.44	(1.18–1.76)
3	365 (28.9)	3105 (24.6)	2.23	(1.85–2.69)
4 (highest)	480 (38.0)	2991 (23.7)	3.06	(2.55–3.66)
Charlson comorbidity index				
0	937 (74.2)	10,344 (82.0)	1.00	1
1	170 (13.5)	1220 (9.7)	1.53	(1.29–1.82)
2 +	155 (12.3)	1056 (8.4)	1.62	(1.35–1.94)
History of estrogen therapy				
No history	1057 (83.8)	10,466 (82.9)	1.00	1
History of estrogen therapy	205 (16.2)	2154 (17.1)	0.94	(0.81–1.10)

### Estrogen therapy exposure and breast cancer mortality risk

Exposure to estrogen therapy with or without concurrent use of AI, tamoxifen, or both, was compared between cases and controls (Table 2). No statistically significant difference in breast cancer mortality was seen in patients using estrogen therapy concurrent to endocrine treatment. Estrogen therapy use without concurrent endocrine treatment was associated with decreased risk for breast cancer mortality.

**Table 2:** Breast cancer mortality risk based on exposure to estrogen therapy

	Cases *N* (%)	Controls *N* (%)	Crude OR (95% CI)	Adjusted * OR (95% CI)	Adjusted** OR (95% CI)
No estrogen therapy	1103 (87.4)	10,850 (86.0)	1	1	1
Estrogen therapy concurrent with AI	48 (3.8)	404 (3.2)	1.17 (0.86–1.58)	0.84 (0.61–1.16)	0.87 (0.62–1.22)
Estrogen therapy concurrent with tamoxifen	44 (3.5)	287 (2.3)	1.50 (1.09–2.08)	1.26 (0.89–1.79)	1.30 (0.91–1.86)
Estrogen therapy concurrent with both AI and tamoxifen as sequential therapy	29 (2.3)	230 (1.8)	1.23 (0.83–1.83)	1.10 (0.73–1.67)	1.14 (0.74–1.74)
Estrogen therapy without concurrent AI or tamoxifen	38 (3.0)	849 (6.7)	0.44 (0.31–0.61)	0.61 (0.43–0.86)	0.61 (0.43–0.87)

*OR* odds ratio, *CI* confidence interval

*Adjusted for age, time from breast cancer diagnosis to index date, T stage, N stage, and chemotherapy

**Adjusted for variables in previous adjustment*, as well as educational level, family income, marital status, CCI, DCI, previous estrogen therapy exposure, region

### Short- and long-term estrogen exposure

To investigate whether estrogen therapy exposure period effects breast cancer mortality, the study cohort exposed to estrogen therapy were divided into two groups; short- and long-term exposure, based on the DDD of estrogen therapy (Table 3). We found no statistically significant differences in breast cancer mortality risk either in short- or long-term estrogen exposure groups concurrent with AI, tamoxifen, or both AI and tamoxifen. Long-term exposure to estrogen therapy without concurrent endocrine treatment was associated with decreased risk for breast cancer mortality.

**Table 3:** Short- and long-term exposure to estrogen therapy and breast cancer mortality risk

	Cases *N* (%)	Controls *N* (%)	Crude OR (95% CI)	Adjusted* OR (95% CI)	Adjusted** OR (95% CI)
No estrogen therapy	1103 (87.4)	10,850 (86.0)	1.00	1.00	1.00
Short-term^#^ estrogen therapy exposure concurrent with AI	25 (2.0)	213 (1.7)	1.15 (0.76–1.75)	0.86 (0.55–1.34)	0.95 (0.61–1.49)
Long-term^#^ estrogen therapy exposure concurrent with AI	23 (1.8)	191 (1.5)	1.18 (0.76–1.83)	0.82 (0.52–1.31)	0.79 (0.49–1.28)
Short-term^#^ estrogen therapy exposure concurrent with tamoxifen	22 (1.7)	156 (1.2)	1.38 (0.88–2.17)	1.28 (0.79–2.08)	1.30 (0.79–2.12)
Long-term^#^ estrogen therapy exposure concurrent with tamoxifen	22 (1.7)	131 (1.0)	1.64 (1.04–2.59)	1.24 (0.76–2.03)	1.31 (0.79–2.15)
Short-term^#^ estrogen therapy exposure concurrent with tamoxifen and AI	20 (1.6)	183 (1.5)	1.07 (0.67–1.71)	0.98 (0.60–1.61)	1.06 (0.64–1.74)
Long-term^#^ estrogen therapy exposure concurrent with tamoxifen and AI	9 (0.7)	47 (0.4)	1.87 (0.91–3.84)	1.53 (0.70–3.38)	1.39 (0.62–3.13)

*OR* odds ratio, *CI* confidence interval

*Adjusted for age, time from breast cancer diagnosis to index date, T stage, N stage and chemotherapy

**Adjusted for variables in previous adjustment*, as well as educational level, family income, marital status, CCI, DCI, previous estrogen therapy exposure, region

#More or less than 90 DDD

## Discussion

This population-based case–control study aimed to investigate potential risks with local estrogen therapy in breast cancer patients with adjuvant endocrine treatment in terms of breast cancer mortality. No statistically significant association between estrogen therapy and breast cancer mortality in patients treated with concurrent tamoxifen, AI, or AI and tamoxifen was observed. The lack of association remained consistent irrespective of the duration of exposure to estrogen therapy.

The study results are in line with the two previous observational studies that did not find an association between local estrogen therapy and breast cancer recurrence or mortality in patients with endocrine treatment [16, 17]. Our study strengthens the current limited evidence with results from a larger cohort with longer follow-up and adds some new insights in this clinically relevant question by presenting separate analyses depending on the type of endocrine treatment and considering the potential impact of short- or long-term exposure to estrogen therapy on the outcome of interest. In fact, Le Ray et al. did not perform a separate analysis for AI users due to lack of adequate data, and they were not able to consider the duration of exposure to estrogen therapy in their analyses. Dew et al. analyzed data from a smaller unselected cohort (1472 women) that included a mixed population of both HR-positive with or without tamoxifen treatment and HR-negative breast cancer, hence, not being able to draw conclusions on possible risks with local estrogen in HR-positive breast cancer patients with concurrent endocrine treatment.

An interesting finding from our analyses was that breast cancer patients exposed to estrogen therapy without concurrent use of endocrine therapy seemed to have a decreased risk for breast cancer mortality. We hypothesize that this patient group might correspond to a low-risk breast cancer group treated with estrogen therapy after the end of adjuvant endocrine treatment and the exposure to estrogen therapy might serve as an indicator of well-being, an association previously described [27].

Our study has several limitations that should be discussed. First, we were not able to distinguish the pharmaceutical form of estrogen therapy. However, as systemic estrogen therapy after breast cancer diagnosis is contraindicated according to the Swedish National Guidelines for breast cancer [29] and this was the case during the time period for study cohort as well, it is reasonable to presume that the vast majority of estrogen therapy prescribed in our study cohort was local estrogen therapy. Further, an unmeasured risk for over-the-counter use of estrogen therapy do exist and should be considered when interpreting our findings. Another potential limitation is the choice of breast cancer mortality as study endpoint. Although breast cancer mortality is a robust and objective outcome, one could argue that the follow-up time is not long enough to investigate mortality as outcome. However, breast cancer recurrence is a less reliable measure than mortality in BCBaSe and choosing breast cancer mortality as endpoint increases the validity of study results. Finally, a causal relationship between exposure and outcome is difficult to be proved with this study design but the dose–response analysis based on exposure to estrogen therapy and the requirement of at least 1 year between exposure to estrogen therapy and mortality are efforts to increase the validity of study results in terms of causality.

In summary, our study did not find any associations between exposure to estrogen therapy and breast cancer mortality in patients treated with tamoxifen or AI, which reassures that local estrogen therapy seems to be safe and can be considered in breast cancer patients with genitourinary symptoms when non-hormonal products are ineffective. Further studies, with more detailed information on prescription patterns and pharmaceutical forms of estrogen therapy, as well as longer follow-up with recurrence as an added endpoint of interest, are warranted to further expand the current evidence.

## Data Availability

The data generated and analyzed during this study are described in the following data record: https://doi.org/10.6084/m9.figshare.1454142340. The Breast Cancer DataBase Sweden (BCBaSe) cohort was used in this study. It is a population-based database that comprises all new cases of invasive breast cancer in women from 1992 to 2012 in three Swedish health care regions. The cohort was linked to a number of national population-based registries. Since BCBaSe contains sensitive health information, it cannot be published in open repositories. Those interested in data from BCBaSe should contact the corresponding author.
